# Effects of SGLT2 inhibitors on cardiac structure and function in stage A and B heart failure with type 2 diabetes: a systematic review and meta-analysis

**DOI:** 10.1186/s40842-026-00306-3

**Published:** 2026-06-23

**Authors:** Vishwani Khanna, Aarushi Batra, Ashok Kumar, Samragyi Juneja, Abhishek Pandeya

**Affiliations:** 1 School of Medical Sciences & Research, Sharda University, Plot No. 32-34, Knowledge Park III, Greater Noida, Uttar Pradesh 201310 India; 2 Santosh University, Ghaziabad, India

**Keywords:** Sodium-Glucose Transporter 2 Inhibitors, Diabetes mellitus, Type 2, Heart failure

## Abstract

**Background:**

Stage A and B heart failure (HF) in type 2 diabetes (T2DM) represents a critical window for prevention. Although SGLT2 inhibitors (SGLT2i) are recommended for early HF risk reduction, their direct structural effects on pre-symptomatic myocardium remain insufficiently characterized in this population.

**Methods:**

MEDLINE, Cochrane Central, Scopus, and ClinicalTrials.gov were searched from inception to February 2026 for studies comparing SGLT2i with placebo in T2DM with Stage A and B HF. Risk of bias was assessed with RoB 2.0. R (version4.5.2) was used to analyse outcomes. Certainty of evidence was assessed using GRADE.

**Results:**

Ten trials (*n* = 773) showed significant reductions in LV mass (–3.37 g), LVMI (–1.84 g/m²) and E/e′ (–0.81). SGLT2i improved weight, BMI, HbA1c, and systolic blood pressure. Safety outcomes were favorable, with no diabetic ketoacidosis reported.

**Conclusions:**

SGLT2i use was associated with a significant reduction in LV mass and improvements in filling pressures, suggesting a favorable modification of underlying myocardium in Stage A and B HF. These changes likely reflect hemodynamic unloading and mass regression rather than immediate functional recovery.

**Supplementary Information:**

The online version contains supplementary material available at 10.1186/s40842-026-00306-3.

**Research Insights**

**Table:** 

Question	Response
What is currently known about this topic?	• Stage A and B HF represents a critical therapeutic window for preventing the transition to symptomatic HF in patients with T2DM. • SGLT2i are established for reducing HF hospitalizations in overt HF, but their specific impact on cardiac remodeling in the subclinical (Stage A and B) T2DM population is less defined. • Current early use evidence is largely extrapolated from macrovascular outcome trials.
What is the key research question?	• Does inhibition of SGLT 2 promote favorable myocardial remodeling in this population?
What is new?	• SGLT2i therapy significantly reduces LV mass and improves diastolic filling pressures. • Favorable remodeling appears to be primarily driven by load reduction rather than restored contractility. • The study provides a focused mechanistic analysis indicating that structural benefits occur even in the absence of symptomatic HF.
How might this study influence clinical practice?	• Findings define the structural substrate for early SGLT2i use to target the structural substrate and possibly attenuate disease progression before overt HF develops. • By identifying load reduction as the primary driver of remodeling, this work helps clinicians better understand the physiological expectations of SGLT2i therapy in primary prevention cohorts.

## Introduction

Heart failure (HF) is widely recognised as a progressive biological continuum, where Stage A (at-risk) and Stage B (pre-clinical structural disease) are the most critical windows for modifying long-term outcomes [1]. The epidemiological concordance between type 2 diabetes (T2DM) and HF is characterized by a bidirectional association where HF typically represents the dominant cardiovascular complication among patients with T2DM, whereas dysglycemia may affect up to 47% of individuals with established HF. This subclinical burden is especially insidious within the T2DM population, with silent structural abnormalities such as impaired global longitudinal strain (GLS), raised left-ventricular (LV) mass and early diastolic dysfunction being seen in up to 40% of asymptomatic individuals across diverse ethnic cohorts [2]. 

These abnormalities lead the pathway to symptomatic HF and carry significant cardiovascular risk, even prior to a decline in ejection fraction (EF). Recognising this, the 2025 ADA Standards of Care provides a Level A recommendation for starting SGLT2 inhibitors early (SGLT2i) in patients with Stage A or B HF [3]. However, the structural effects of SGLT2i on pre-symptomatic myocardium remain insufficiently characterised. Current recommendations are largely extrapolated from landmark cardiovascular outcome trials (CVOTs) such as EMPA-REG OUTCOME, CANVAS, DECLARE-TIMI 58 and VERTIS-CV that were powered for macrovascular events and hospitalisations rather than direct measures of favorable myocardial remodeling [4–7]. Consequently, essential structural parameters, including GLS, LV mass, LV mass index (LVMI), or E/e′ have seldom been evaluated in this cohort.

This evidentiary gap raises an important mechanistic query: does SGLT2 inhibition mediate favorable myocardial remodeling in Stage A and B HF or are its clinical benefits a direct consequence of systemic metabolic and hemodynamic optimisation? Distinguishing durable structural remodeling from transient diuretic-mediated “unloading” is key in pre-symptomatic state, when minor changes might be mistakenly attributed to variations in volume status. Hence, characterizing these drivers is key to defining the biological foundation for early SGLT2i therapy in this population.

The current evidence base includes small mechanistic randomised controlled trials (RCTs) utilizing sensitive imaging modalities speckle tracking echocardiography or cardiac magnetic resonance imaging [CMR]). When viewed as individual studies, these are often underpowered and heterogeneous. To address this gap, we performed a systematic review and meta-analysis of RCTs assessing SGLT2i only in Stage A and B HF groups. Our aim was to examine the effect of SGLT2i therapy on cardiac structure and function (cardiac biomarkers, echocardiogram parameters), associated metabolic changes and safety profile. By isolating the pre-symptomatic cohort, this analysis clarifies whether SGLT2i confer true structural benefit in this population, providing a dedicated mechanistic synthesis for their early clinical use.

## Methods

Our meta-analysis adhered to the Cochrane Handbook for Systematic Reviews of Interventions and was reported following the Preferred Reporting Items for Systematic Reviews and Meta-analysis (PRISMA) guidelines [8, 9]. This review is registered with the International Prospective Register of Systematic Reviews (PROSPERO) under the identifier CRD420261304023. Ethical approval was not necessary for our study.

### Eligibility criteria

Eligible studies were RCTs enrolling adults with T2DM classified as Stage A (at risk for HF) or Stage B (structural heart disease without symptoms). Trials were included if they evaluated any SGLT2i in comparison with placebo or standard care. To ensure relevance to early HF biology, studies were required to report at least one structural or functional cardiac outcome. Included trials were categorized into Stage A or Stage B HF based on the 2022 ACC/AHA/HFSA Universal Definition. Stage A trials included patients at risk (T2DM) without structural heart disease, while Stage B trials required evidence of structural heart disease or biomarker elevation in asymptomatic individuals.

#### Outcomes

Primary outcomes

- Cardiac structural and functional parameters: GLS, LV mass, LVMI, LVEF, and E/e′ measured by echocardiography or CMR.

Secondary outcomes

- Metabolic and anthropometric measures: HbA1c, weight, BMI, systolic blood pressure (SBP).
- Safety outcomes: incidence of hypoglycaemia, diabetic ketoacidosis (DKA), urinary tract infections (UTI), and genital infections.

### Information sources

We conducted searches across various databases and international trial registers from their inception to February 2026, without imposing any language limitations. The sources included the Cochrane Central Register of Controlled Trials, MEDLINE (via PubMed), Scopus, and ClinicalTrials.gov. Additionally, we reviewed the reference lists of included articles and relevant reviews to identify additional potential studies. Supplementary Table 1 shows a comprehensive overview of our search strategy.

### Selection process

All articles obtained from our online search were deduplicated and screened. After deduplication, two authors individually reviewed titles and abstracts. The remaining articles were subject to thorough full-text screening by two authors independently. Any discrepancies were resolved by a third reviewer. Detailed PICO selection criteria are listed in Supplementary Table 2.

### Data collection process

Following the process of study selection, two reviewers extracted data into a pre-piloted Excel spreadsheet. The extracted data items included patient characteristics, intervention details, study characteristics, and outcome variables. For studies with incomplete reporting of outcomes, we attempted to contact the original investigators for clarification. In cases where no response was received, analyses were conducted using all available data reported in the primary publication.

### Risk of bias (RoB) assessment

We assessed RoB at the study level using the updated Cochrane Risk of Bias tool for randomised trials (RoB 2.0). Two authors independently judged each trial as low, high, or some concerns. Any discrepancies were resolved by a third reviewer.

### Data synthesis

All statistical analyses were performed using R (version 4.5.2). Continuous outcomes were synthesized as mean differences (MDs) with 95% confidence intervals (CIs). For trials reporting data as median and interquartile range (IQR), means and SD were estimated using the methods of Wan et al. [10]. This transformation was specifically applied to systolic blood pressure in LEAVE-DM; weight and BMI in ELUCIDATE and EMPACEF; HbA1c in DAPA-LVH; and LV mass and LVMI in EMPACEF.

When reported, change‑from‑baseline means and standard deviations (SD) were used preferentially assuming a correlation coefficient of 0.5 for change-from-baseline calculations. All data transformations were performed programmatically in R to ensure consistency and reproducibility. Inter‑study heterogeneity was assessed using the restricted maximum likelihood (REML) method and quantified using the I² statistic. Given anticipated clinical and methodological variability across trials, random‑effects models were prespecified as the primary analytic approach.

Heterogeneity for direct comparisons was evaluated by the I² statistic, with thresholds of 0–40% (possibly unimportant), 30–60% (moderate), 50–90% (substantial) and 75–100% (considerable). We used funnel plots to evaluate publication bias visually and Egger’s test when ten or more studies were available.

We performed subgroup analyses to investigate possible contributors of heterogeneity. We assessed if the risk of bias and imaging modality would modify the observed effects. Subgroup analyses were conducted only if each subgroup had two or more studies. An interaction p-value of less than 0.05 was interpreted as significant.

### Certainty of evidence assessment

We assessed the certainty of evidence using the Grading of Recommendations, Assessment, Development, and Evaluation (GRADE) framework. The pooled estimates were characterised as high, moderate, low, or very low quality [11]. 

## Results

### Search results

Our initial search produced 1834 search results. After deduplication and title and abstract screening, 64 studies were included for full-text screening. The detailed study selection process is represented in Supplementary Fig. 1.

### Characteristics of included studies

Our meta‑analysis included ten RCTs enrolling a total of 773 participants across Europe, Asia, Australia, and North America [12–21]. Trials evaluated dapagliflozin, empagliflozin, or ertugliflozin at approved therapeutic doses, with treatment durations ranging from 3 to 12 months. The mean age of participants across studies ranged from the mid‑50s to early 70s. Baseline BMI values, when reported, generally fell within the overweight to obese range. Cardiac imaging modalities included both echocardiography and CMR, with four studies using CMR and six using echocardiography. Of the 10 included trials, three enrolled participants classified as Stage A HF while seven targeted Stage B populations with evidence of structural remodeling or dysfunction. Across all ten trials, baseline concomitant therapies—including metformin, other glucose-lowering agents, ACEi/ARB, beta-blockers, calcium-channel blockers, diuretics, and statins—were comparable between intervention and control groups, with no significant between-group differences.

Supplementary Table 3 lists the detailed characteristics of included studies and participants and Supplementary Table 4 lists the specific diagnostic criteria utilized by each included trial.

### Risk of bias (RoB)

Apart from the ELUCIDATE trial (Lin et al., 2024), which was classified as having a high RoB, all included studies were judged to be at low RoB. The primary driver of bias for this study was lack of blinding. Supplementary Fig. 2 provides an overview of our risk of bias evaluation.

### Primary outcomes (cardiac structural and functional outcomes)

**Fig. 1:**
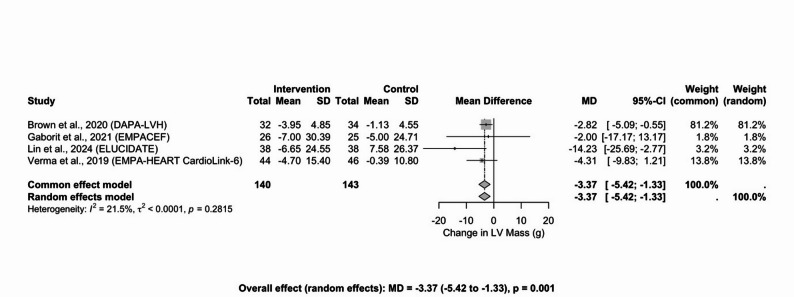
: Effect of SGLT2i on LV mass

**Fig. 2:**
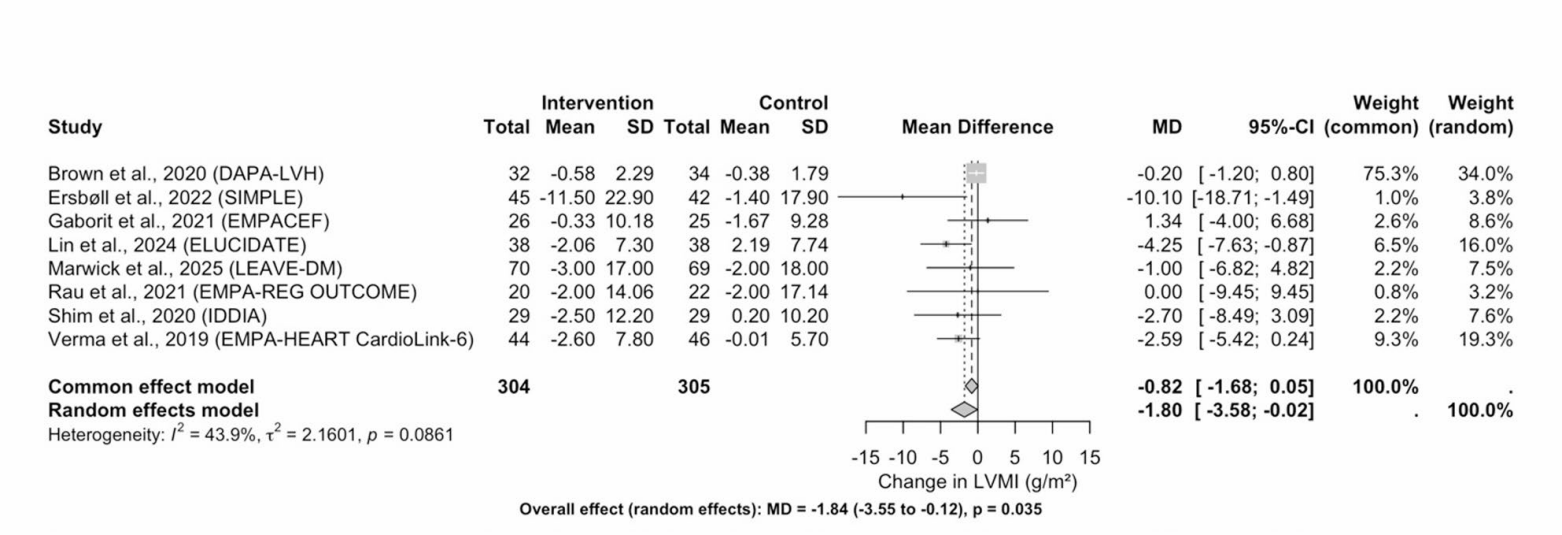
: Effect of SGLT2i on LVMI

**Fig. 3:**
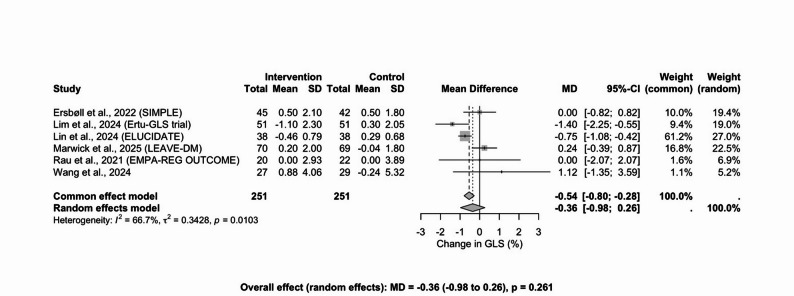
: Effect of SGLT2i on GLS

**Fig. 4:**
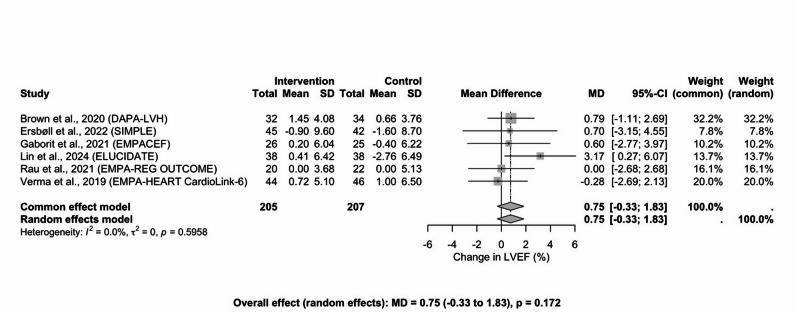
: Effect of SGLT2i on LVEF

**Fig. 5:**
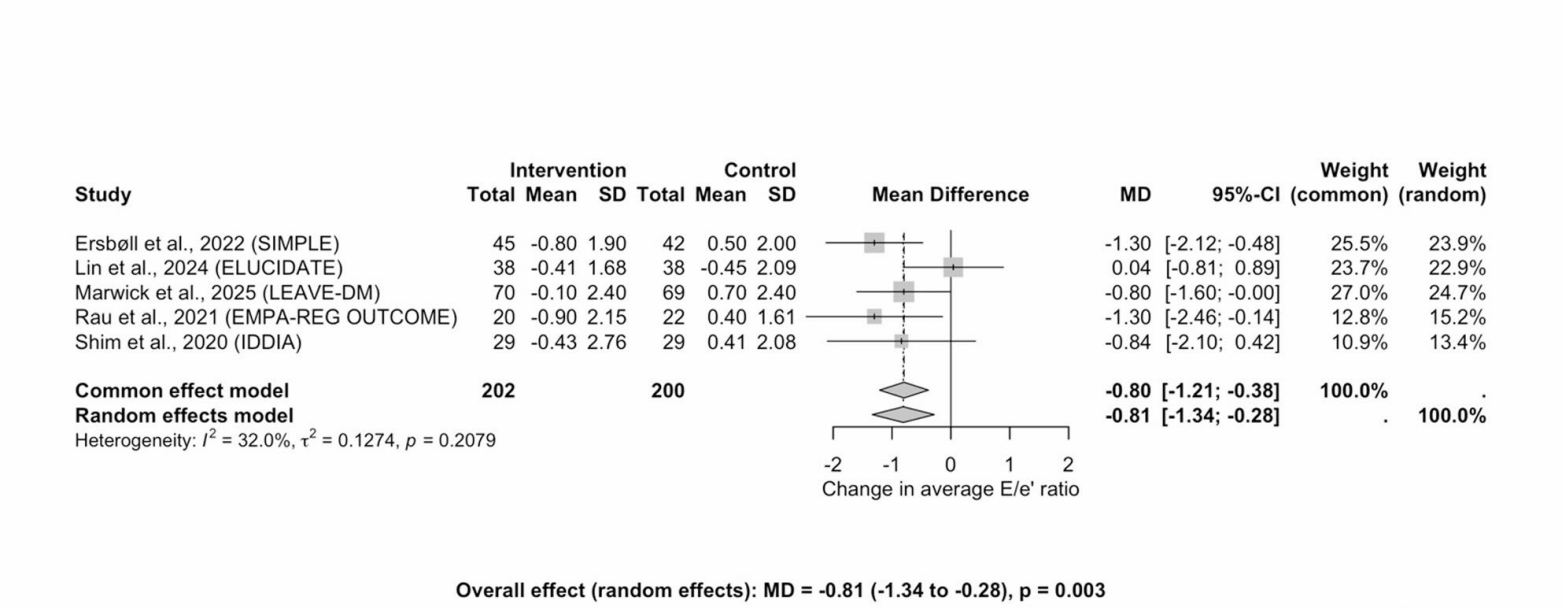
: Effect of SGLT2i on E/e′ ratio

1. LV Mass: Four trials reported LV mass by cardiac MRI. The pooled mean difference (MD) was − 3.37 g (95% CI − 5.42 to − 1.33; I² = 21.5%; P-value = 0.001; Fig. 1).

2. LVMI: Eight trials reported LVMI. The pooled MD was − 1.84 g/m² (95% CI − 3.55 to − 0.12; I² = 43.9%; P-value = 0.035; Fig. 2).

3. GLS: Six trials reported GLS. The pooled MD was − 0.36% (95% CI − 0.98 to 0.26; I² = 66.7%; P-value = 0.261; Fig. 3).

4. LVEF: Six trials reported LVEF. The pooled MD was 0.75% (95% CI − 0.33 to 1.83; I² = 0%; P-value = 0.172; Fig. 4).

1. E/e′ Ratio: Five trials reported E/e′. The pooled MD was − 0.81 (95% CI − 1.34 to − 0.28; I² = 32.0%; P-value = 0.003; Fig. 5).

### Secondary outcomes (metabolic and anthropometric outcomes)

**Fig. 6:**
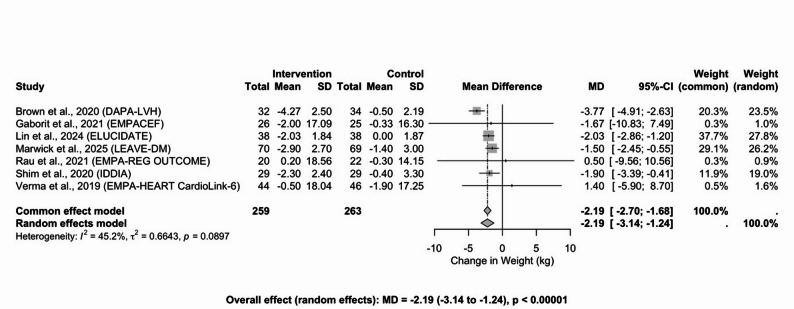
: Effect of SGLT2i on body weight

**Fig. 7:**
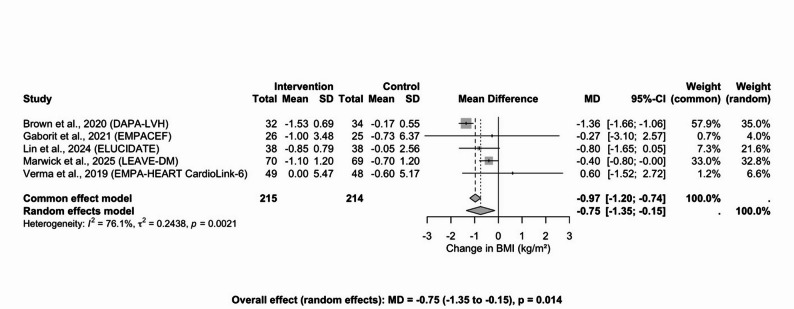
: Effect of SGLT2i on BMI

**Fig. 8:**
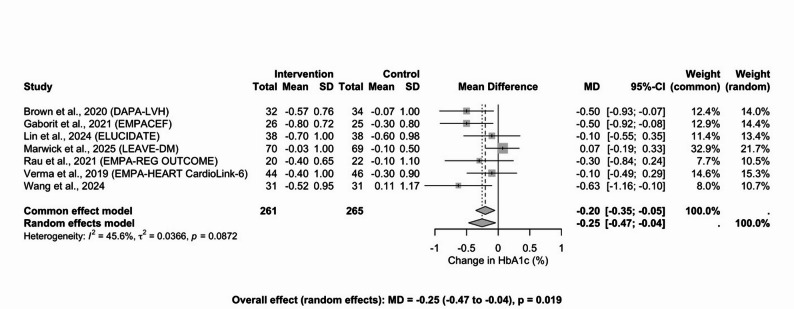
: Effect of SGLT2i on HbA1c

**Fig. 9:**
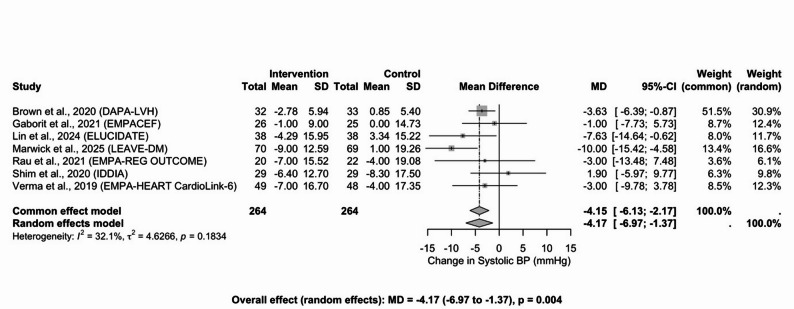
: Effect of SGLT2i on SBP

6. Body Weight: Seven trials reported weight change. The pooled MD was − 2.19 kg (95% CI − 3.14 to − 1.24; I² = 45.2%; P-value < 0.00001; Fig. 6).

7. BMI: Five trials reported BMI. The pooled MD was − 0.75 kg/m² (95% CI − 1.35 to − 0.15; I² = 76.1%; P-value = 0.014; Fig. 7).

8. HbA1c: Seven trials reported HbA1c. The pooled MD was − 0.25% (95% CI − 0.47 to − 0.04; I² = 45.6%; P-value = 0.019; Fig. 8).

9. SBP: Seven trials reported SBP. The pooled MD was − 4.17 mmHg (95% CI − 6.97 to − 1.37; I² = 32.1%; P-value = 0.004; Fig. 9).

The certainty of evidence for primary cardiac outcomes ranged from low to high. We observed High-quality evidence for the reduction in LV mass (MD − 3.37 g) and E/e′ ratio (MD − 0.81), with no serious limitations in risk of bias, inconsistency, or imprecision. The evidence for LVMI was graded as Moderate, downgraded once due to substantial heterogeneity (I^2^ = 43.9%). Evidence for LVEF was also Moderate, downgraded for imprecision as the 95% CI crossed the null. The certainty for GLS was rated as Low, downgraded for both substantial heterogeneity (I^2^ = 66.7%) and imprecision (Table 1).

**Table 1:** Integrated summary of the pooled effect estimates alongside the corresponding GRADE assessments of evidence certainty

Continuous outcome	N*	Risk of bias	Heterogeneity	Indirectness	Imprecision	Publication bias	Overall quality	MD (95% CI)
BMI (kg/m²)	5	No serious limitation	Serious limitation§	No serious limitation	No serious limitation¶	No serious limitation†	Moderate	−0.75 (− 1.35, − 0.15)
Body weight (kg)	7	No serious limitation	Serious limitation§	No serious limitation	No serious limitation¶	No serious limitation†	Moderate	−2.19 (− 3.14, − 1.24)
HbA1c (%)	7	No serious limitation	Serious limitation§	No serious limitation	No serious limitation¶	No serious limitation†	Moderate	−0.25 (− 0.47, − 0.04)
SBP (mmHg)	7	No serious limitation	No serious limitation§§	No serious limitation	No serious limitation¶	No serious limitation†	High	−4.17 (− 6.97, − 1.37)
LV mass (g)	4	No serious limitation	No serious limitation§§	No serious limitation	No serious limitation¶	No serious limitation†	High	−3.37 (− 5.42, − 1.33)
LVMI (g/m²)	8	No serious limitation	Serious limitation§	No serious limitation	No serious limitation¶	No serious limitation†	Moderate	−1.84 (− 3.55, − 0.12)
LVEF (%)	6	No serious limitation	No serious limitation§§	No serious limitation	Serious limitation**	No serious limitation†	Moderate	0.75 (− 0.33, 1.83)
GLS	6	No serious limitation	Serious limitation§	No serious limitation	Serious limitation**	No serious limitation†	Low	−0.36 (− 0.98, 0.26)
E/e′ ratio	5	No serious limitation	No serious limitation§§	No serious limitation	No serious limitation¶	No serious limitation†	High	−0.81 (− 1.34, − 0.28)

* = Number of included studies

§ Quality was downgraded due to heterogeneity as the I² statistic indicated substantial heterogeneity

§§ Quality was not downgraded due to heterogeneity as the I² statistic was in the potentially unimportant range

¶ Quality was not downgraded due to imprecision as the 95% CI did not cross the line of no effect

** Quality was downgraded due to imprecision as the 95% CI crossed the line of no effect

† Quality was not downgraded due to publication bias since fewer than 10 studies were available and no strong evidence suggested missing negative studies

### Safety outcomes

The safety profile of SGLT2i across the included trials was consistent with established clinical data, characterised by a low absolute incidence of serious adverse events (SAEs). Due to the high frequency of zero-event arms, safety data were synthesized qualitatively rather than through formal meta-analysis. Detailed event rates by study are provided in Supplementary Table 7.

Clinical Events and Mortality Clinical progression was rare. Progression to symptomatic HF (AHA Stage C/D) occurred in only one patient in the SGLT2i cohort. All-cause mortality was zero across all trials reporting this metric.

Specific adverse events of interest were recorded as follows:

- Hypoglycemia: The most frequently reported event, occurring in seven patients (SGLT2i: *n* = 7; Placebo: *n* = 2). The majority of these events were clustered in the Lim et al. (2024) and Rau et al. (2021) cohorts.
- Infections: Rates of UTI were low (SGLT2i: *n* = 4; Placebo: *n* = 9). Genital infections occurred in six participants in the SGLT2i and one in the placebo group.
- DKA: There were zero reported cases of DKA across all trial arms, totalling 515 patient observations.

### Heterogeneity and subgroup analysis

To explore the sources of statistical heterogeneity observed in our primary analysis, we performed pre-specified subgroup analyses based on imaging modality (CMR vs. Echocardiography) and a sensitivity analysis based on RoB. In accordance with our protocol, subgroup analyses were only conducted for outcomes featuring at least two studies per stratum. A summary of subgroup analysis is given in Supplementary Table 8 A and 8B.

#### Impact of imaging modality

Subgroup analysis by imaging modality was feasible for LVMI. A significant subgroup difference was observed (P-interaction = 0.049; Supplementary Fig. 3).

- Echocardiography: Studies utilizing echocardiography demonstrated a significant reduction in LVMI with no heterogeneity (MD: -3.60 g/m^2^; 95% CI: -6.02 to -1.19; P-value = 0.003; I^2^ = 0%).
- CMR: In contrast, the pooled effect among studies using the gold-standard CMR was not statistically significant with low heterogeneity (MD: -0.66 g/m^2^; 95% CI: -2.32 to 1.01; P-value = 0.363; I^2^ = 30%).

This suggests that the choice of imaging modality may influence the magnitude of detected structural changes, with echocardiography potentially overestimating the treatment effect compared to CMR.

#### RoB and sensitivity analysis

A sensitivity analysis was performed by excluding the only RCT judged to have high RoB, ELUCIDATE trial (Lin et al., 2024) to assess the stability of the primary outcomes.

The significant reductions in LV Mass (MD: -3.02 g; 95% CI: -5.10 to -0.94), E/e’ ratio (MD: -1.06; 95% CI: -1.53 to -0.58), and Body Weight (MD: -2.21 kg; 95% CI: -3.54 to -0.88) remained robust and statistically significant (*p* < 0.01 for all). However, the effects on LVMI (*p* = 0.13) and BMI (*p* = 0.11) were no longer statistically significant after the exclusion of the high-RoB study. Furthermore, statistical heterogeneity remained moderate to substantial for GLS (I2 = 63.5%) and BMI (I2 = 81.9%), suggesting that RoB was not the primary driver of the observed inconsistency for these specific markers. (Supplementary Figs. 4–11)

## Discussion

This systematic review and meta-analysis provides a dedicated synthesis of SGLT2i effects on the pre-symptomatic (Stage A and B) diabetic myocardium. Our findings demonstrate that SGLT2i therapy induces a significant reduction in LV mass and an improvement in diastolic filling pressures (E/e’), suggesting a tangible structural benefit that precedes the onset of symptomatic HF. However, the absence of a significant effect in GLS suggests that the effects of SGLT2i in this early stage are more likely driven by improvements in hemodynamic loading conditions, specifically the reduction in preload and afterload, as well as LV mass regression rather than an immediate restoration of intrinsic myocardial contractility.

The absence of significant change in LVEF aligns with the baseline characteristics of the included cohorts, who predominantly exhibited preserved systolic function. Given that LVEF is more reflective of later stages of myocardial failure, its relative stability in this subclinical population supports the interpretation that SGLT2i benefits at this stage are largely directed towards attenuating adverse remodeling, rather than acutely increasing contractile fraction. Importantly, the neutral results for GLS, a more sensitive marker of subclinical systolic dysfunction, warrant careful interpretation. While preclinical models suggest that SGLT2i may enhance myocyte contractility through calcium handling, our quantitative synthesis suggests that in the human Stage A and B population, the predominant mechanism of action of SGLT2i occurs via hemodynamic load reduction (afterload and preload), rather than a direct inotropic effect on the myocardium.

The observed reduction in LV mass (-3.37 g) and LVMI (-1.84 g/m²) is a pivotal finding, as LV hypertrophy is a potent predictor of progression to clinical HF and therefore, this regression likely represents a critical interruption of the HF continuum [22]. The concomitant improvement in E/e’ (MD: -0.81; *p* = 0.003) without significant changes in GLS or LVEF suggests that in the early stages of the HF continuum, SGLT2i act predominantly by mitigating ventricular “stiffness” and reducing hemodynamic afterload. The consistent reduction in systolic blood pressure (-4.17 mmHg) likely acts synergistically with direct metabolic pathways to drive this regression. Thus, while SGLT2i therapy effectively targets the structural substrate of diabetic cardiomyopathy, the timeline for possible functional systolic recovery may extend beyond the 3–12-month follow-up periods observed in current RCTs.

The biological substrate for this favorable remodeling is likely multifactorial. Beyond systemic hemodynamic offloading, SGLT2i appear to exert direct cardioprotective effects by inhibiting the myocardial sodium-hydrogen exchanger (NHE-1), thereby normalizing intracellular calcium signalling and reducing oxidative stress [23]. Furthermore, a shift toward ketone body utilization optimizes myocardial energy efficiency in the bioenergetically compromised diabetic heart [24]. Importantly, the observed reduction in E/e’ and LV mass may also reflect a mitigation of pro-fibrotic signalling and subclinical inflammation, effectively delaying or possibly arresting the transition from metabolic derangement to irreversible structural damage [25]. 

A critical finding of our analysis is the discrepancy in LVMI results based on imaging modality (P-interaction = 0.049). While echocardiography-based trials suggested robust reductions (MD: -3.60 g/m²; P-value = 0.003), the pooled effect among studies utilizing the gold standard CMR was not statistically significant (MD: -0.66 g/m²; P-value = 0.363). This interaction suggests that the magnitude of parenchymal change may have been over-estimated by the geometric assumptions of echocardiography, which are sensitive to the diuretic and natriuretic effects of SGLT2i. CMR, being volume-independent, provides a more conservative but arguably more accurate assessment of true parenchymal change. For clinicians, this underscores that while echocardiography remains an essential screening tool, the exact magnitude of mass regression in the context of SGLT2i should be interpreted with caution.

The 2025 ADA/ACC/AHA “Level A” recommendations for Stage A and B populations are primarily supported by the prevention of clinical events. Our findings provide a mechanistic link by demonstrating that these clinical benefits are accompanied by measurable, albeit subtle, improvements in cardiac architecture. These results reinforce that the benefits of SGLT2i extend beyond glucose-lowering to actively modify the structural substrate of the heart during the most treatable phases of the disease.

One of the strong points of our review is its methodological rigor, by strictly following PRISMA guidelines and including only RCTs for evidence sources. We also utilised a thorough prespecified analytic framework including a random effects model and multiple sensitivity and subgroup analyses to investigate potential sources of heterogeneity. By isolating trials with only Stage A and B HF in this population, we provide a “clean” mechanistic view of SGLT2i without the confounding influence of advanced HF or intensive polypharmacy. However, several limitations warrant consideration. The analysis is limited by relatively small sample size. Furthermore, the short follow-up durations (3–12 months) are likely insufficient to capture the full trajectory of myocardial recovery, particularly of functional markers such as GLS which may require more prolonged intervention to demonstrate meaningful improvement. The moderate-to-substantial heterogeneity observed in GLS suggests that this population is phenotypically diverse and the response to therapy may be modulated by the degree of baseline metabolic derangement. Finally, while structural markers are vital surrogates, long-term longitudinal data are required to confirm that these improvements translate into a durable delay in the transition to symptomatic Stage C HF.

## Conclusion

In Stage A and B HF with T2DM, SGLT2i use was associated with significant reductions in LV mass and improvements in diastolic function. The lack of significant changes in GLS and LVEF likely reflects short follow-up duration, suggesting that benefits are driven primarily by hemodynamic unloading rather than immediate myocardial recovery. These findings provide a biological foundation for the early initiation of therapy, as recommended by contemporary guidelines, to possibly attenuate the progression of structural disease before the onset of clinical symptoms. Future long-term trials should be conducted to explore these parameters more comprehensively.

## Electronic Supplementary Material

Below is the link to the electronic supplementary material.

Supplementary Material 1

## Data Availability

All extracted data and analyses generated for this meta-analysis are included in the article and its Supplementary Information. The original trial data are publicly available in the published studies cited in this review.
