# Supplementary material for: Effects of SGLT2 inhibitors on cardiac structure and function in stage A and B heart failure with type 2 diabetes: a systematic review and meta-analysis
Source: Cardiovasc Diabetol Endocrinol Rep. 2026 Jun 23;12:45. doi: 10.1186/s40842-026-00306-3 (PMC13288799; doi:10.1186/s40842-026-00306-3)
Supplement: Supplementary file 1 — Supplementary Material 1 [file 40842_2026_306_MOESM1_ESM.docx]

Table of Contents

[Supplementary Table 1 : Search strategy for databases 3](#_Toc224740903)

[Supplementary Table 2 : PICO Criteria 5](#_Toc224740904)

[Supplementary Figure 1 : PRISMA flowchart 5](#_Toc224740905)

[Supplementary Table 3 : Baseline characteristics of included studies 7](#_Toc224740906)

[Supplementary Table 4 : Study‑specific eligibility criteria for Stage A and Stage B heart failure across included RCTs 7](#_Toc224740907)

[Supplementary Table 5 : Baseline Concomitant Therapies Across Included RCTs 9](#_Toc224740908)

[Supplementary Table 6 : Baseline cardiac structural and functional parameters across included RCTs 10](#_Toc224740909)

[Supplementary Figure 2 : Risk of bias assessment of included studies visualised using robvis tool 11](#_Toc224740910)

[Supplementary Table 7 : Summary of reported safety outcomes across included RCTs 12](#_Toc224740911)

[Supplementary Table 8B : Subgroup Analysis by Imaging Modality 13](#_Toc224740912)

[Supplementary Figure 3 : LVMI subgroup analysis by imaging method 15](#_Toc224740913)

[Supplementary Figure 4 : Sensitivity analysis for LVMI excluding high-RoB study. 15](#_Toc224740914)

[Supplementary Figure 5 : Sensitivity analysis for LV mass excluding high-RoB study. 16](#_Toc224740915)

[Supplementary Figure 6 : Sensitivity analysis for average E/e’ ratio excluding high-RoB study. 17](#_Toc224740916)

[Supplementary Figure 7 : Sensitivity analysis for GLS excluding high-RoB study. 18](#_Toc224740917)

[Supplementary Figure 8 : Sensitivity analysis for SBP excluding high-RoB study. 19](#_Toc224740918)

[Supplementary Figure 9 : Sensitivity analysis for body weight excluding high-RoB study. 20](#_Toc224740919)

[Supplementary Figure 10 : Sensitivity analysis for HbA1c excluding high-RoB study. 21](#_Toc224740920)

[Supplementary Figure 11 : Sensitivity analysis for BMI excluding high-RoB study. 22](#_Toc224740921)

[References 22](#_Toc224740922)

| **Database** | **Number** | **Search terms** | **Records retrieved** |
| --- | --- | --- | --- |
| PubMed | #1 | dapagliflozin[tiab] OR empagliflozin[tiab] OR canagliflozin[tiab] OR ertugliflozin[tiab]    OR ipragliflozin[tiab] OR luseogliflozin[tiab] OR tofogliflozin[tiab] OR sotagliflozin[tiab]    OR "Sodium-Glucose Transporter 2 Inhibitors"[Mesh] OR SGLT2[tiab] | 17,613 |
|  | #2 | "diabetic cardiomyopathy"[tiab] OR ("diabetes mellitus"[Mesh] AND cardiomyopathy[tiab])    OR "left ventricular hypertrophy"[tiab] OR "diastolic dysfunction"[tiab] | 37,312 |
|  | #3 | "Heart Failure"[Mesh] OR "heart failure"[tiab] OR "cardiac remodeling"[tiab] OR "ventricular dysfunction"[tiab] | 319,270 |
|  | #4 | #1 AND #2 AND #3 | 239 |
| Scopus | #5 | TITLE-ABS-KEY ( dapagliflozin OR empagliflozin OR canagliflozin OR ertugliflozin OR ipragliflozin OR luseogliflozin OR tofogliflozin OR sotagliflozin OR SGLT2 OR "sodium glucose cotransporter 2" ) | 36,488 |
|  | #6 | TITLE-ABS-KEY ( "diabetic cardiomyopathy" OR ( diabetes AND cardiomyopathy ) OR "left ventricular hypertrophy" OR "diastolic dysfunction" ) | 67,468 |
|  | #7 | TITLE-ABS-KEY ( "heart failure" OR "cardiac remodeling" OR "ventricular dysfunction" ) | 512,581 |
|  | #8 | #5 AND #6 AND #7 | 1,461 |
| Cochrane Central Register of Controlled Trials | #9 | (dapagliflozin OR empagliflozin OR canagliflozin OR ertugliflozin OR ipragliflozin   OR luseogliflozin OR tofogliflozin OR sotagliflozin OR SGLT2 OR "sodium glucose cotransporter 2") | 7172 |
|  | #10 | ("diabetic cardiomyopathy" OR (diabetes AND cardiomyopathy)  OR "left ventricular hypertrophy" OR "diastolic dysfunction") | 3515 |
|  | #11 | ("heart failure" OR "cardiac remodeling" OR "ventricular dysfunction") | 42797 |
|  | #12 | #9 AND #10 AND #11 | 134 |
| Clinical trials.gov | #13 | Condition/Disease: “diabetic cardiomyopathy” OR “diabetes mellitus AND cardiomyopathy” OR “left ventricular hypertrophy” OR “diastolic dysfunction” OR “heart failure” OR “cardiac remodeling” OR “ventricular dysfunction” OR “diabetes mellitus AND heart failure” AND Intervention/Treatment: dapagliflozin OR empagliflozin OR canagliflozin OR ertugliflozin OR ipragliflozin OR luseogliflozin OR tofogliflozin OR sotagliflozin OR “SGLT2 inhibitor” | 276 |

# Supplementary Table 1 : Search strategy for databases

| **Domain** | **Criteria** |
| --- | --- |
| Population | Adults (≥18 years) with Type 2 Diabetes Mellitus (T2DM)  Classified as Stage A or B Heart Failure (at risk for HF or structural heart disease without current/prior symptoms) |
| Intervention | Sodium-glucose cotransporter-2 inhibitors (SGLT2i), including empagliflozin, dapagliflozin, canagliflozin, or ipragliflozin. |
| Comparator | Placebo |
| Primary Outcomes | Cardiac structural and functional parameters measured using echo or cardiac magnetic resonance imaging |
| Secondary Outcomes | Metabolic and anthropometric parameters |
| Study Design | Randomised controlled trials |
| Exclusion Criteria | Non‑randomized studies, observational designs, case series, case reports  Studies without a placebo comparator  History of or current AHA Stage C and D HF  Uncontrolled hypertension (Mean systolic blood pressure (BP) >180 mmHg or diastolic BP >95 mmHg after three separate measurements Impaired renal function, defined as estimated glomerular filtration rate (eGFR  < 45 ml/min/1.73 m2 of body-surface-area or end-stage renal failure or Dialysis. Animal studies, pediatric populations, or non‑cardiac surgery |

# Supplementary Table 2 : PICO Criteria


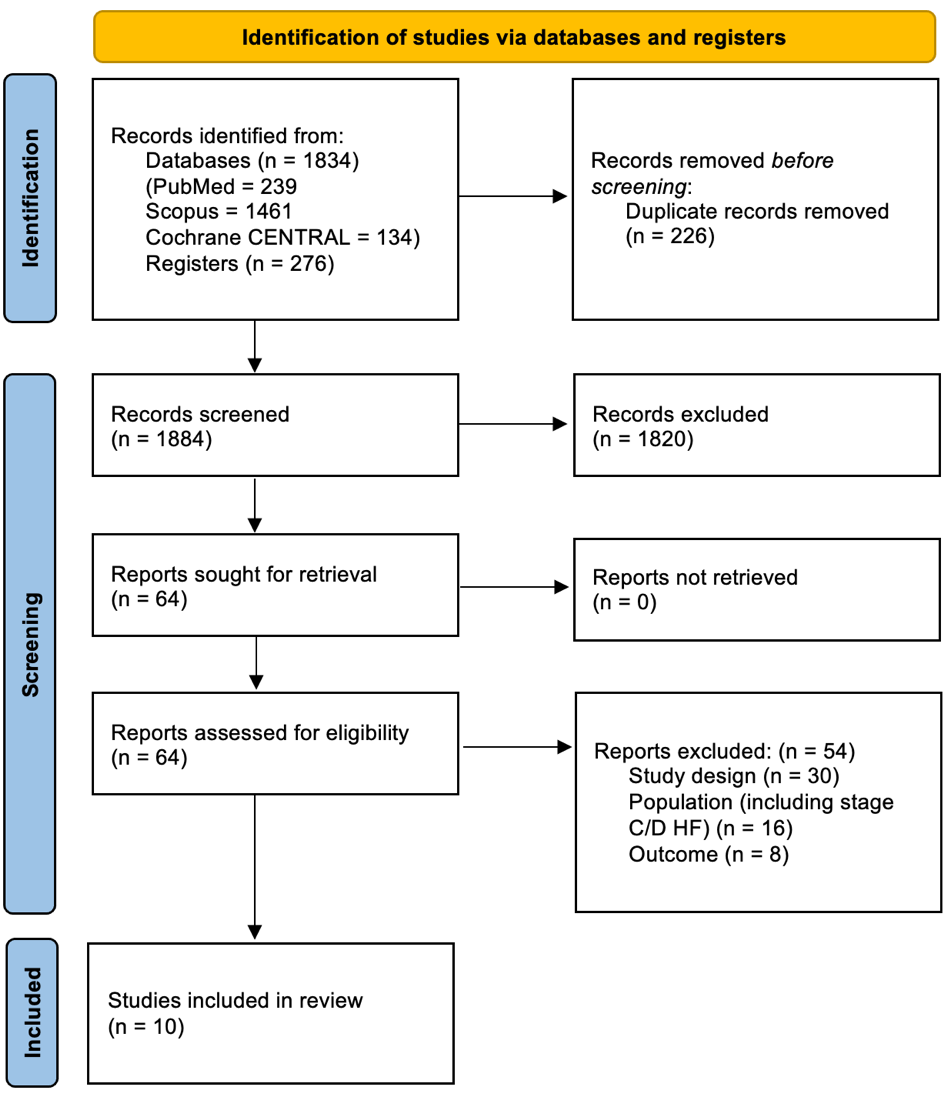


# Supplementary Figure 1 : PRISMA flowchart

| **Study ID** | **Trial ID** | **Country** | **SGLT 2** | **Dose (mg/day)** | **Duration (Months)** | **Number** | | **Age (Mean ± SD)** | | **Male %** | | **BMI (Mean ± SD)** | | **Imaging method** |
| --- | --- | --- | --- | --- | --- | --- | --- | --- | --- | --- | --- | --- | --- | --- |
|  |  |  |  |  |  | **I** | **P** | **I** | **P** | **I** | **P** | **I** | **P** |  |
| Brown et al., 2020 (DAPA-LVH) [1] | NCT02956811 | Scotland | Dapagliflozin | 10 | 12 | 32 | 34 | 64.25 ± 7.01 | 66.74 ± 6.62 | 62.5 | 52.9 | 32.30 ± 4.66 | 32.59 ± 4.22 | CMR |
| Ersbøll et al., 2022 (SIMPLE) [2] | NCT03151343 | Denmark | Empagliflozin | 25 | 3.25 | 45 | 42 | 66.2 ± 9.2 | 66.7 ± 8.9 | 75.6 | 85.7 | 31.8 ± 6.4 | 29.1 ± 3.8 | ECHO |
| Gaborit et al., 2021 (EMPACEF) [3] | NCT03118336 | France | Empagliflozin | 10 | 3 | 26 | 25 | 57.0 ± 10.1 | 58.6 ± 9.2 | 38 | 40 | 33.6 ± 3.7 | 34.7 ± 7.2 | CMR |
| Lim et al., 2024 (Ertu-GLS trial) [4] | NCT03717194 | South Korea | Ertugliflozin | 5 | 6 | 51 | 51 | 62.5 ± 9.9 | 65.3 ± 8.4 | NR | NR | 26.3 ± 2.9 | 26.4 ± 3.6 | ECHO |
| Lin et al., 2024 (ELUCIDATE) [5] | NCT03871621 | Taiwan | Dapagliflozin | 10 | 6 | 38 | 38 | 54.32 ± 11.05 | 58.5 ± 9.02 | 45 | 40 | 27.04 ± 5.19 | 25.71 ± 3.35 | ECHO |
| Marwick et al., 2025 (LEAVE-DM) [6] | ACTRN12619001393145 | Australia | Dapagliflozin | 10 | 6 | 70 | 69 | 73 ± 5 | 73 ± 5 | 61.4 | 61 | 30 ± 5 | 32 ± 6 | ECHO |
| Rau et al., 2021 (EMPA-REG OUTCOME) [7] | 2016-000172-19 | Germany | Empagliflozin | 10 | 3 | 20 | 22 | 62.8 ± 5.4 | 61.2 ± 7.9 | 80 | 81.8 | 31.4 ± 5.3 | 31.2 ± 4.0 | ECHO |
| Shim et al., 2020 (IDDIA) [8] | NCT02751398 | South Korea | Dapagliflozin | 10 | 6 | 29 | 29 | NR | NR | NR | NR | NR | NR | ECHO |
| Verma et al., 2019 (EMPA-HEART CardioLink-6 ) [9] | NCT02998970 | Canada | Empagliflozin | 10 | 6 | 44 | 46 | NR | NR | NR | NR | NR | NR | CMR |
| Wang et al., 2024 [10] | NCT03782259 | USA | Dapagliflozin | 10 | 12 | 31 | 31 | 62 ± 9 | 62 ± 11 | 81 | 84 | NR | NR | CMR |

*I = Intervention; P = Placebo; CMR = Cardiac magnetic resonance imaging*

# Supplementary Table 3 : Baseline characteristics of included studies

| **Study ID** | **HF Stage** | **Study Specific Eligibility & Diagnostic Criteria** |
| --- | --- | --- |
| Brown et al., 2020 (DAPA-LVH) [1] | Stage B | T2DM + Left Ventricular Hypertrophy (LVH) confirmed by MRI (LV mass index >95 g/m² in men, >77 g/m² in women) or Echo. |
| Ersbøll et al., 2022 (SIMPLE) [2] | Stage B | T2DM + established structural heart disease or high-risk cardiovascular disease (prior MI, CABG, or PCI) without HF symptoms. |
| Gaborit et al., 2021 (EMPACEF) [3] | Stage A | T2DM without known structural heart disease, history of CAD, or symptoms of heart failure. |
| Lim et al., 2024 (Ertu-GLS trial) [4] | Stage B | T2DM + "Pre-HF" defined by structural disease (LVH: LVMI >115/95 g/m²), dysfunction (GLS < 18% or e' < 7 cm/s), or NT-proBNP >125 pg/mL. |
| Lin et al., 2024 (ELUCIDATE) [5] | Stage A | T2DM with LVEF ≥50% and no clinical history of heart failure or structural heart disease. |
| Marwick et al., 2025 (LEAVE-DM) [6] | Stage B | T2DM + asymptomatic subclinical LV dysfunction (defined by low Global Longitudinal Strain or abnormal E/e’ ratio). |
| Rau et al., 2021 (EMPA-REG OUTCOME) [7] | Stage B | T2DM + established atherosclerotic cardiovascular disease (structural risk) with no prior diagnosis of heart failure. |
| Shim et al., 2020 (IDDIA) [8] | Stage B | T2DM + objective Diastolic Dysfunction (e' < 8 cm/s) without clinical signs or symptoms of heart failure. |
| Verma et al., 2019 (EMPA-HEART CardioLink-6 ) [9] | Stage B | T2DM + stable Coronary Artery Disease (structural heart disease) with preserved LVEF and no diagnosis of heart failure. |
| Wang et al., 2024 [10] | Stage A | T2DM for ≥1 year without known heart failure, structural heart disease, or chronic kidney disease. |

# Supplementary Table 4 : Study‑specific eligibility criteria for Stage A and Stage B heart failure across included RCTs

| **Study ID** | **Metformin (I/P)** | **Sulfonylurea (I/P)** | **DPP‑4i (I/P)** | **GLP‑1RA (I/P)** | **TZD (I/P)** | **AGI (I/P)** | **Insulin (I/P)** | **ACEi/ARB (I/P)** | **BB (I/P)** | **CCB (I/P)** | **Diuretic (I/P)** | **Statin (I/P)** | **Notes** |
| --- | --- | --- | --- | --- | --- | --- | --- | --- | --- | --- | --- | --- | --- |
| Brown et al., 2020 (DAPA‑LVH) | 100 vs 100 | 21.9 vs 23.5 | 12.5 vs 8.8 | 12.5 vs 8.8 | 0 vs 8.8 | NR | 21.9 vs 20.6 | 53.1 vs 52.9 (ACEi); 15.6 vs 17.6 (ARB) | 12.5 vs 14.7 | 28.1 vs 38.2 | 28.1 vs 11.8 | 78.1 vs 88.2 | All differences NS |
| Ersbøll et al., 2022 (SIMPLE) | 77.8 vs 85.7 | 6.7 vs 4.8 | 13.3 vs 23.8 | 26.7 vs 26.2 | NR | NR | 57.8 vs 57.1 | 84.4 vs 71.4 | 57.8 vs 33.3 | NR | NR | NR | BB numerically higher in EMPA arm; NS |
| Gaborit et al., 2021 (EMPACEF) | NR | NR | NR | NR | NR | NR | NR | NR | NR | NR | NR | NR | Authors state baseline therapies balanced |
| Lim et al., 2024 (Ertu‑GLS) | 84.3 vs 82.4 | 21.6 vs 17.6 | 21.6 vs 23.5 | 0 vs 0 | 3.9 vs 5.9 | 2.0 vs 0 | 9.8 vs 11.8 | 43.1 vs 47.1 | 17.6 vs 13.7 | 21.6 vs 25.5 | 11.8 vs 9.8 | 49.0 vs 47.1 | All differences NS |
| Lin et al., 2024 (ELUCIDATE) | 26.3 vs 34.2 | 31.6 vs 31.6 | 73.7 vs 63.2 | 0 vs 0 | 15.8 vs 10.5 | 13.2 vs 7.9 | 28.9 vs 23.7 | NR | NR | NR | NR | 76.3 vs 68.4 | All differences NS |
| Marwick et al., 2025 (LEAVE‑DM) | 84.3 vs 73.9 | NR | NR | 0 vs 0 | NR | NR | 11.4 vs 8.7 | 68.6 vs 75.4 | 27.1 vs 26.1 | NR | NR | 78.6 vs 72.5 | All differences NS |
| Rau et al., 2021 (EMPA-REG OUTCOME) [7] | 65 vs 82 | NR | 40 vs 27 | NR | NR | NR | 55 vs 36 | 75 vs 91 | 80 vs 73 | 20 vs 23 | 50 vs 45 | 75 vs 68 |  |
| Shim et al., 2020 (IDDIA) | NR | NR | NR | NR | NR | NR | NR | NR | NR | NR | NR | NR | Authors state baseline therapies balanced |
| Verma et al., 2019 (EMPA‑HEART CardioLink‑6) | 96 vs 92 | NR | NR | NR | NR | NR | 25 vs 25 | 82 vs 85 | 78 vs 81 | 12 vs 31 | 4 vs 13 | 96 vs 96 | All differences NS |
| Wang et al., 2024 | 84 vs 84 | NR | NR | NR | NR | NR | 39 vs 36 | ACEi 42 vs 45; ARB 29 vs 32 | 16 vs 26 | 26 vs 23 | NR | NR | All differences NS |

*All between‑group differences were reported as non‑significant (NS) in the original publications.*

***Abbreviations:*** *I = Intervention; P = Placebo; DPP‑4i = Dipeptidyl Peptidase‑4 inhibitor; GLP‑1RA = Glucagon‑Like Peptide‑1 Receptor Agonist; TZD = Thiazolidinedione; AGI = Alpha‑Glucosidase Inhibitor; ACEi/ARB = Angiotensin‑Converting Enzyme inhibitor / Angiotensin Receptor Blocker; BB = Beta-blocker; CCB = Calcium Channel Blocker; NR = Not reported; NS = Non‑significant.*

# Supplementary Table 5 : Baseline Concomitant Therapies Across Included RCTs

| **Trial** | **LV Mass (g) (I/P)** | **LVMI (g/m²) (I/P)** | **LVEF (%) (I/P)** | **LVEDV (mL) (I/P)** | **LVESV (mL) (I/P)** | **LAVI (mL/m²) (I/P)** | **E/e′ (I/P)** | **GLS (%) (I/P)** |
| --- | --- | --- | --- | --- | --- | --- | --- | --- |
| Brown et al., 2020 (DAPA‑LVH) | 126.47 / 121.61 | 60.92 / 59.04 | 71.31 / 72.54 | 127.63 / 120.66 | 37.17 / 33.63 | NR | NR | NR |
| Ersbøll et al., 2022 (SIMPLE) | NR | 102 / 96.5 | 56.6 / 58.2 | 83.4 / 83.8 | 36.5 / 35.6 | 35.3 / 32.5 | 10.5 / 8.7 | −16.5 / −17.0 |
| Gaborit et al., 2021 (EMPACEF) | 129 / 117 | 64 / 58 | 63.7 / 63.7 | NR | NR | NR | NR | NR |
| Lim et al., 2024 (Ertu‑GLS trial) | NR | NR | NR | NR | NR | NR | NR | −15.5 / −16.7 |
| Lin et al., 2024 (ELUCIDATE) | 133.85 / 129.36 | 37.62 / 36.52 | 62.72 / 63.48 | 90.18 / 86.17 | 34.04 / 31.35 | NR | 8.41 / 9.00 | −18.69 / −18.49 |
| Marwick et al., 2025 (LEAVE‑DM) | NR | 88 / 88 | 58 / 58 | NR | NR | 36 / 35 | 10.3 / 9.8 | −17.1 / −16.9 |
| Rau et al., 2021 (EMPA-REG OUTCOME) [7] | NR | 86/91 | 41/48 | 49/50 | 34/36 | 28/31 | 9.1/9.3 | -19/-17 |
| Shim et al., 2020 (IDDIA) | NR | 94.2 / 93.6 | NR | NR | NR | 28.6 / 27.6 | 11.08 / 9.70 | NR |
| Verma et al., 2019 (EMPA‑HEART CardioLink‑6) | 116.5 / 120.9 | 59.3 / 62.2 | 58.0 / 55.5 | 124.1 / 138.4 | 53.0 / 62.5 | NR | NR | NR |
| Wang et al., 2024 | NR | NR | NR | NR | NR | NR | NR | −12.9 / −11.0 |

***Abbreviations****: I = Intervention; P = Placebo; LV Mass = left ventricular mass; LVMI = left ventricular mass index; LVEF = left ventricular ejection fraction; LVEDV = left ventricular end‑diastolic volume; LVESV = left ventricular end‑systolic volume; LAVI = left atrial volume index; E/e′ = ratio of early mitral inflow velocity to mitral annular early diastolic velocity; GLS = global longitudinal strain.*

# Supplementary Table 6 : Baseline cardiac structural and functional parameters across included RCTs


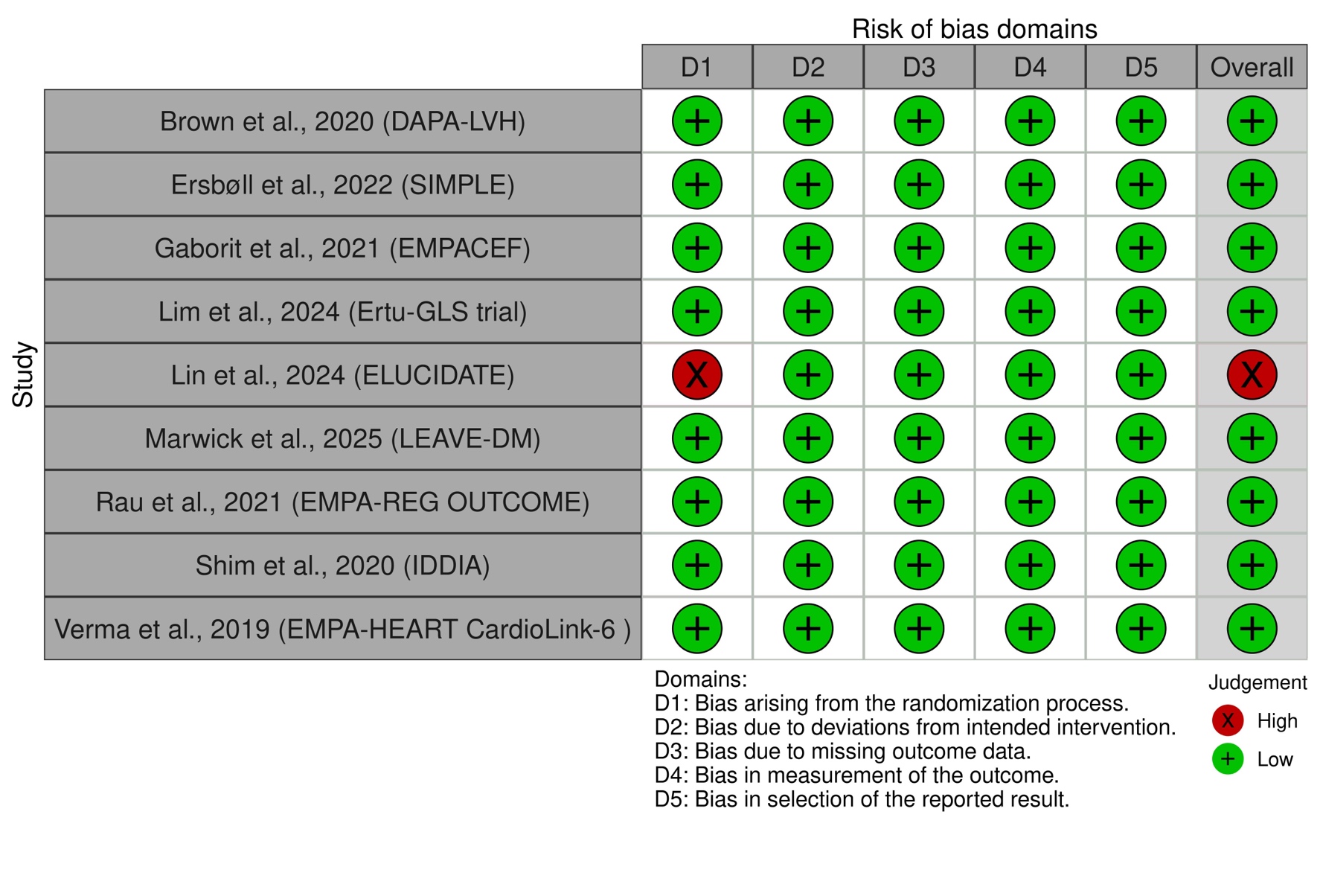


# Supplementary Figure 2 : Risk of bias assessment of included studies visualised using robvis tool

McGuinness, LA, Higgins, JPT. Risk-of-bias VISualization (robvis): An R package and Shiny web app for visualizing risk-of-bias assessments. Res Syn Meth. 2020; 1-7. [https://doi.org/10.1002/jrsm.1411](https://www.google.com/url?q=https://doi.org/10.1002/jrsm.1411&sa=D&source=editors&ust=1768812218565912&usg=AOvVaw23qiXOFmQKzvuVUou_5w_d)

| **Study ID** | **Number (I/P)** | **Symptomatic HF incidence (AHA stage C/D) (I/P)** | **All-cause mortality (I/P)** | **Hypoglycemia (I/P)** | **Genital infection (I/P)** | **UTI (I/P)** | **DKA (I/P)** |
| --- | --- | --- | --- | --- | --- | --- | --- |
| Brown et al., 2020 (DAPA-LVH) | 32/34 | NR | NR | NR | NR | 1/6 | 0/0 |
| Lim et al., 2024 (Ertu-GLS trial) | 51/51 | NR | NR | 3/1 | 2/1 | 2/3 | 0/0 |
| Lin et al., 2024 (ELUCIDATE) | 38/38 | 0/0 | NR | 0/0 | 0/0 | 0/0 | 0/0 |
| Marwick et al., 2025 (LEAVE-DM) | 70/69 | 1/0 | NR | NR | NR | NR | NR |
| Rau et al., 2021 (EMPA-REG OUTCOME) | 20/22 | NR | 0/0 | 4/1 | 4/0 | 1/0 | 0/0 |
| Verma et al., 2019 (EMPA-HEART CardioLink-6 ) | 44/46 | NR | 0/0 | NR | NR | NR | 0/0 |

***Abbreviations****: I = Intervention; P = Placebo; HF= Heart failure; UTI = Urinary tract infection; DKA =Diabetic ketoacidosis*

# Supplementary Table 7 : Summary of reported safety outcomes across included RCTs

| **Outcome** | **Primary Analysis MD (95% CI)** | **Sensitivity Analysis MD (95% CI)*** | **Primary I^2^** | **Sensitivity I^2^** |
| --- | --- | --- | --- | --- |
| LVMI (g/m2) | -1.84 (-3.55, -0.12) | -1.19 (-2.76, 0.37) | 41.50% | 23.10% |
| LV Mass (g) | -3.37 (-5.42, -1.33) | -3.02 (-5.10, -0.94) | 21.50% | 0.00% |
| GLS (%) | -0.36 (-0.98, 0.26) | -0.20 (-1.00, 0.60) | 66.70% | 63.50% |
| E/e' Ratio | -0.81 (-1.34, -0.28) | -1.06 (-1.53, -0.58) | 32.00% | 0.00% |
| Weight (kg) | -2.19 (-3.14, -1.24) | -2.21 (-3.54, -0.88) | 45.20% | 53.40% |
| BMI (kg/m2) | -0.75 (-1.35, -0.15) | -0.68 (-1.50, 0.15) | 76.10% | 81.80% |
| HbA1c (%) | -0.25 (-0.47, -0.04) | -0.28 (-0.53, -0.04) | 45.60% | 53.70% |
| SBP (mmHg) | -4.17 (-6.97, -1.37) | -3.68 (-6.82, -0.54) | 32.10% | 35.90% |

**Sensitivity analysis performed by excluding Lin et al., 2024 (ELUCIDATE)*

Supplementary Table 8A : Sensitivity Analysis (Excluding High Risk of Bias Study)

| **Outcome** | **Subgroup** | **No. of Studies** | **MD (95% CI)** | **I2** | **P-interaction** |
| --- | --- | --- | --- | --- | --- |
| LVMI (g/m2) | Echocardiography | 5 | -3.60 (-6.02, -1.19) | 0.00% | 0.048 |
|  | Cardiac MRI | 3 | -0.71 (-2.25, 0.82) | 19.10% |  |

# Supplementary Table 8B : Subgroup Analysis by Imaging Modality


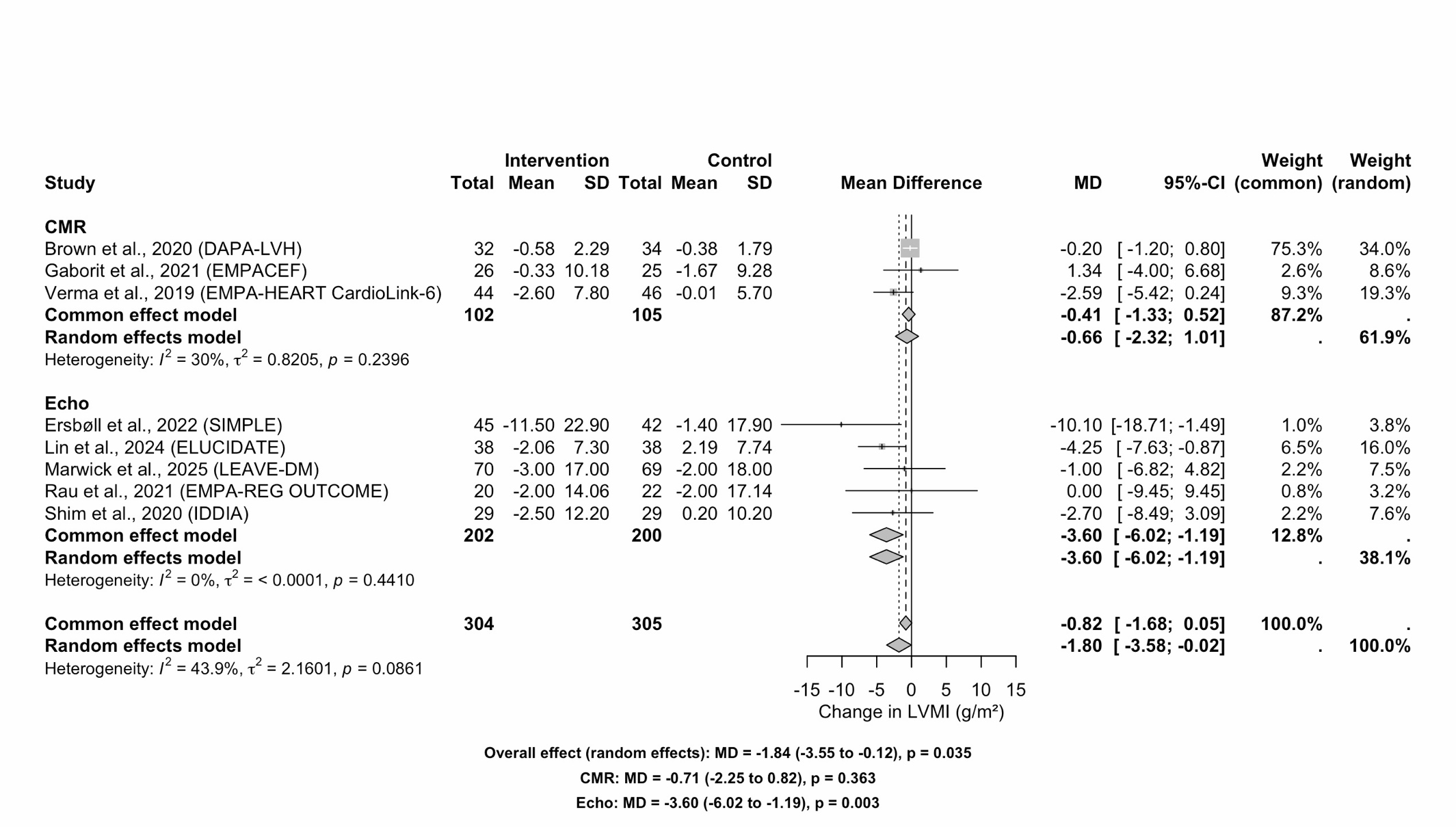


# Supplementary Figure 3 : LVMI subgroup analysis by imaging method


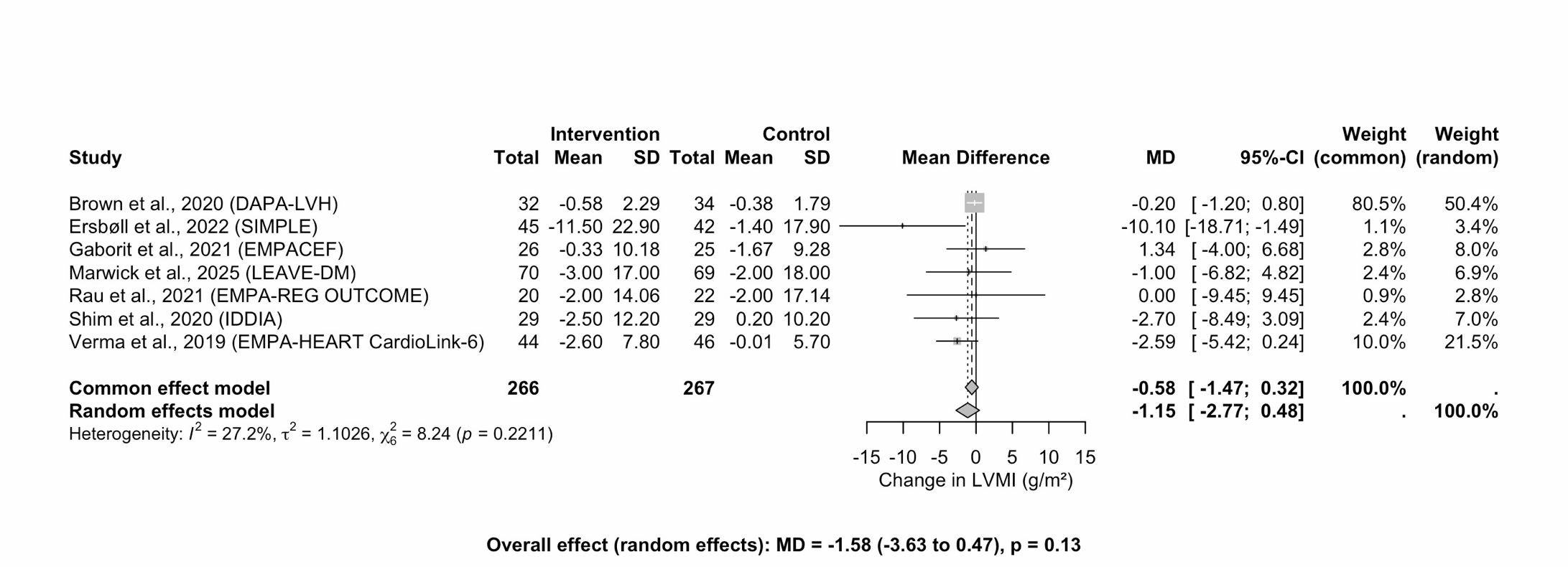


Supplementary Figure 4 : Sensitivity analysis for LVMI excluding high-RoB study.


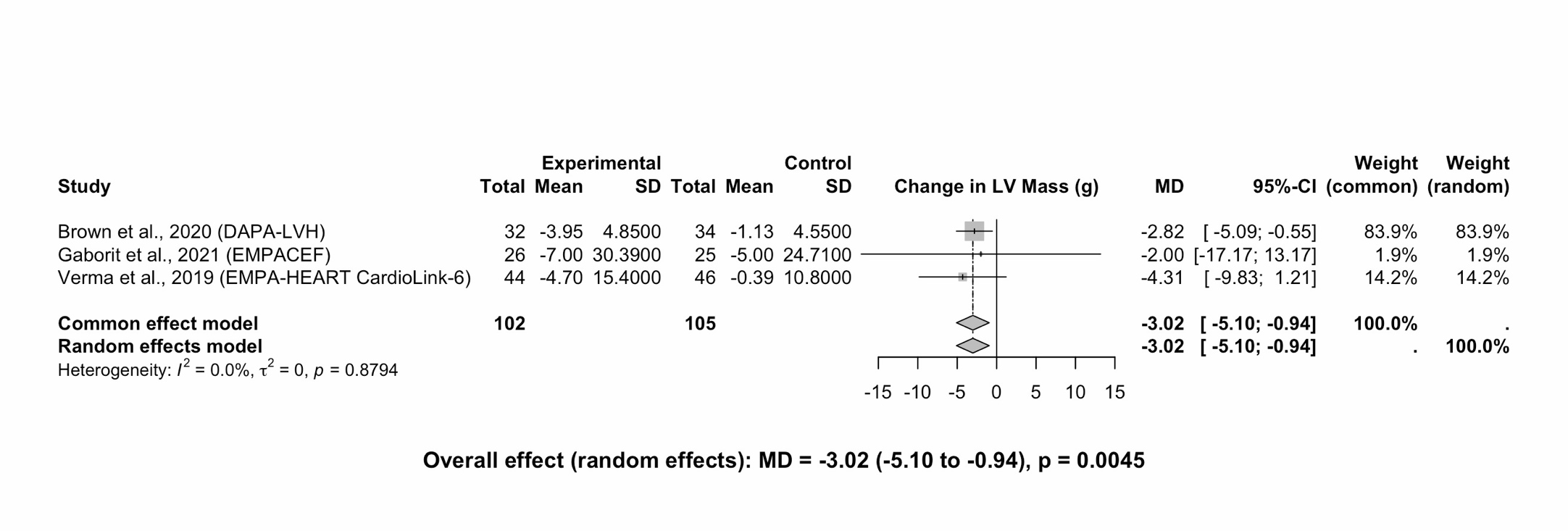


Supplementary Figure 5 : Sensitivity analysis for LV mass excluding high-RoB study.


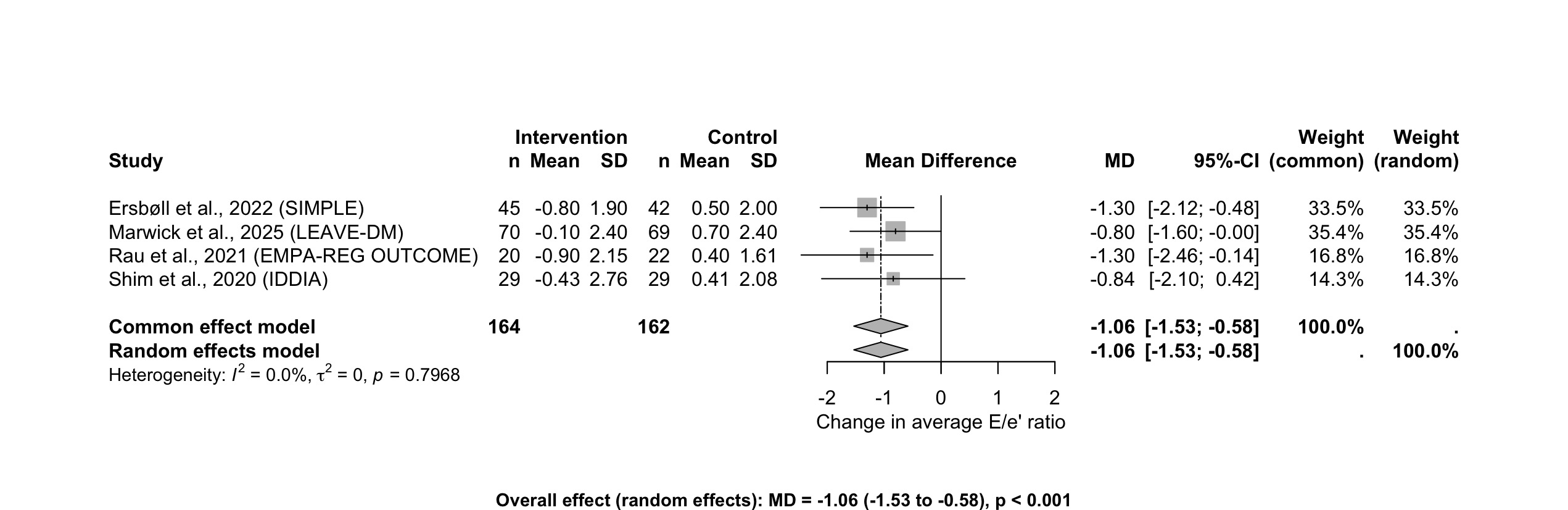


Supplementary Figure 6 : Sensitivity analysis for average E/e’ ratio excluding high-RoB study.


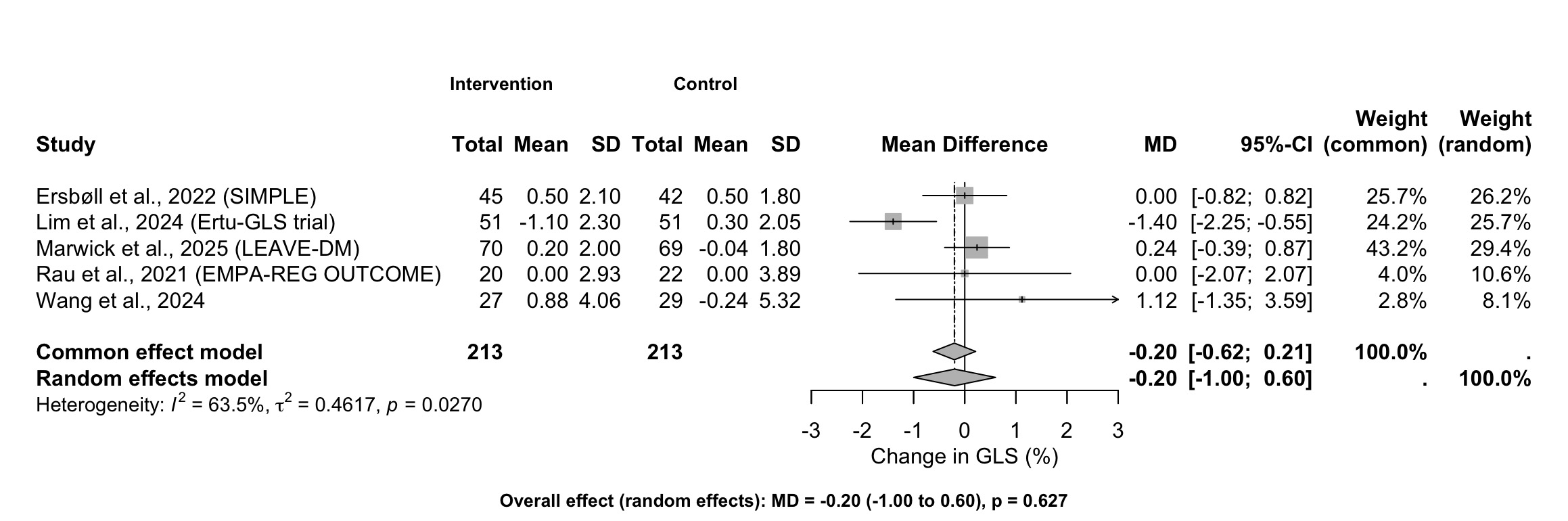


Supplementary Figure 7 : Sensitivity analysis for GLS excluding high-RoB study.


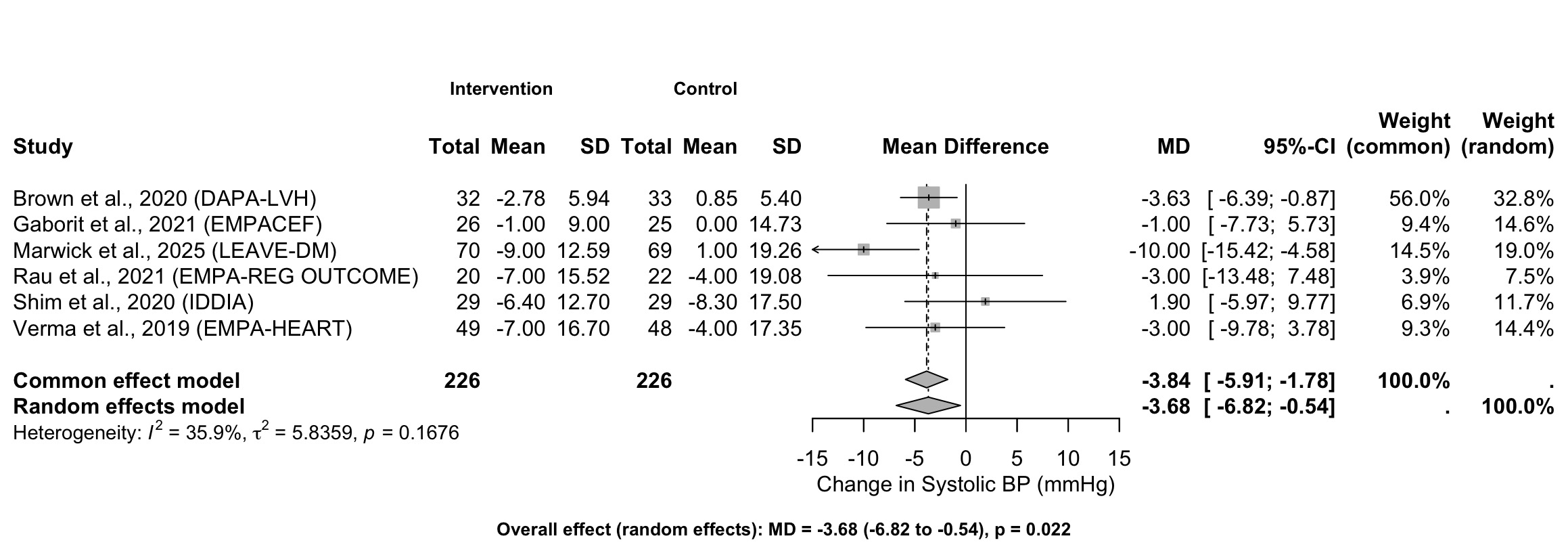


Supplementary Figure 8 : Sensitivity analysis for SBP excluding high-RoB study.


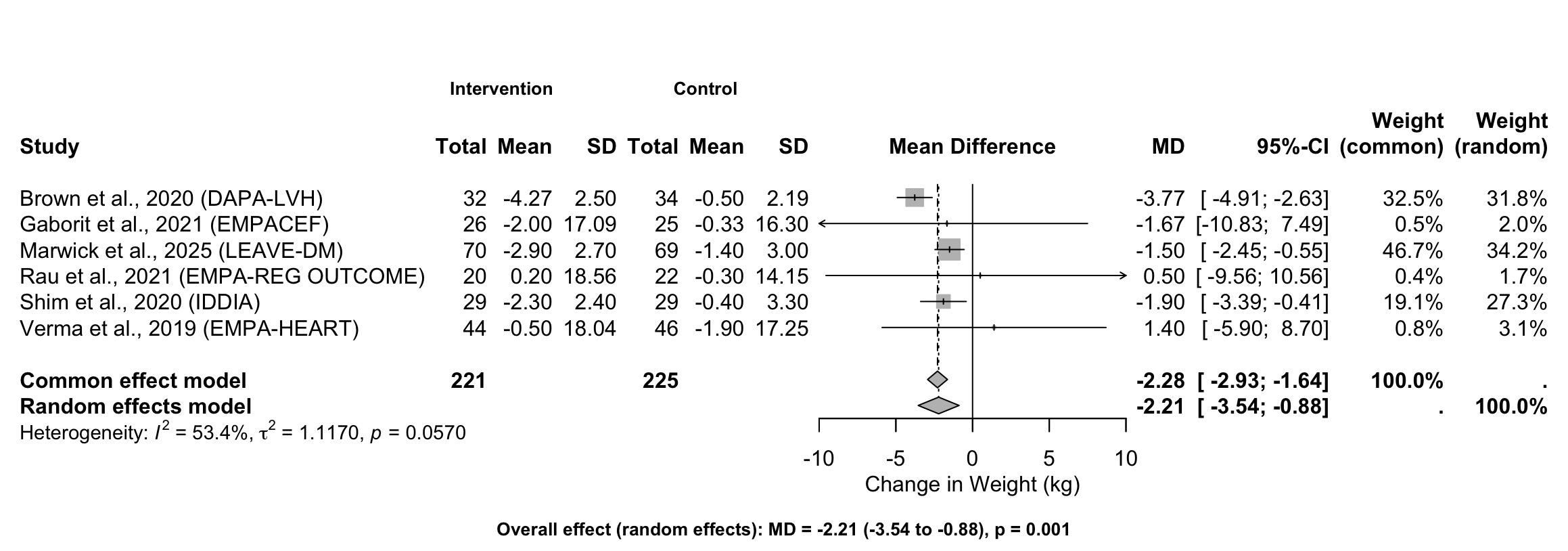


Supplementary Figure 9 : Sensitivity analysis for body weight excluding high-RoB study.


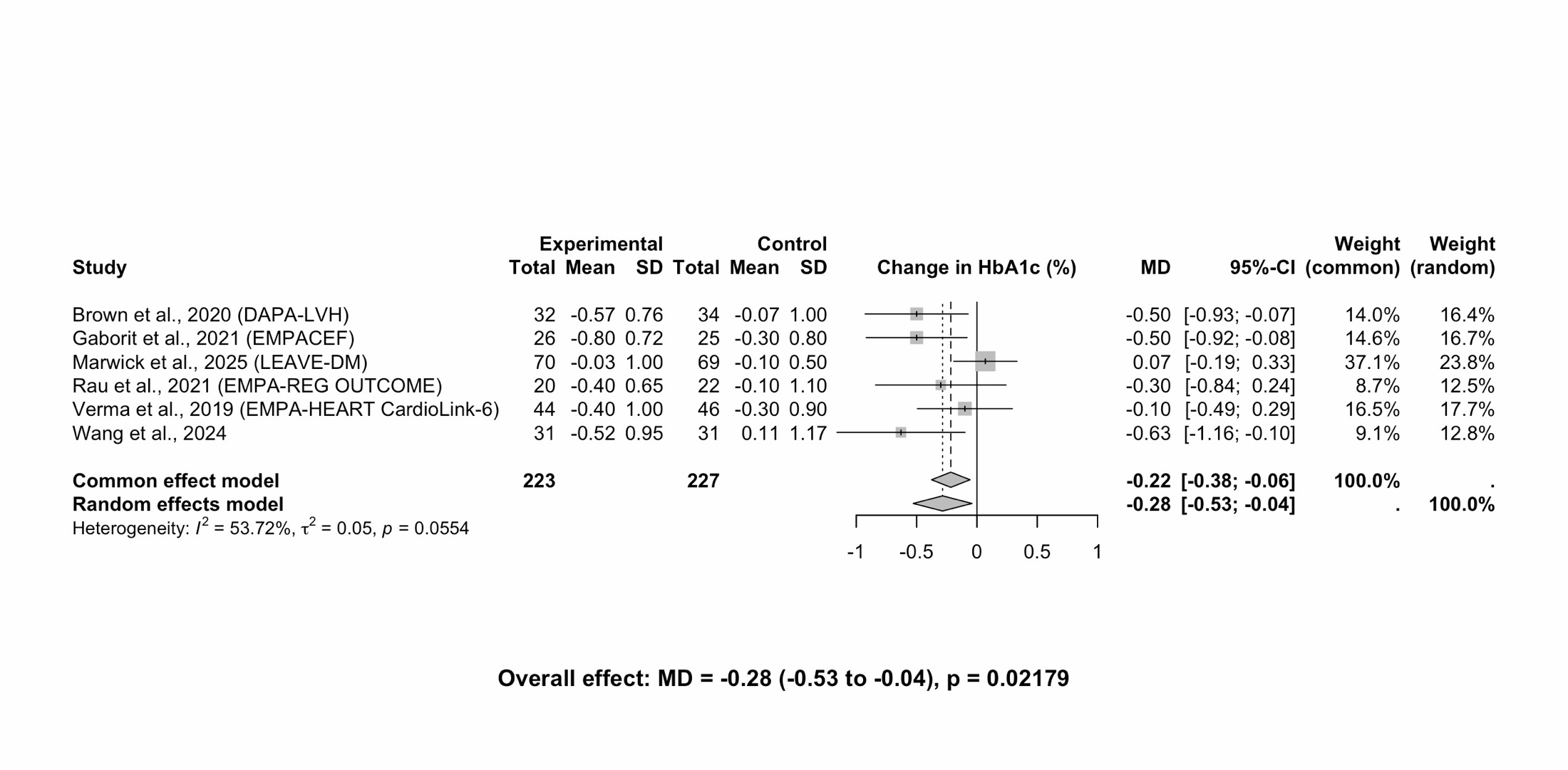


Supplementary Figure 10 : Sensitivity analysis for HbA1c excluding high-RoB study.


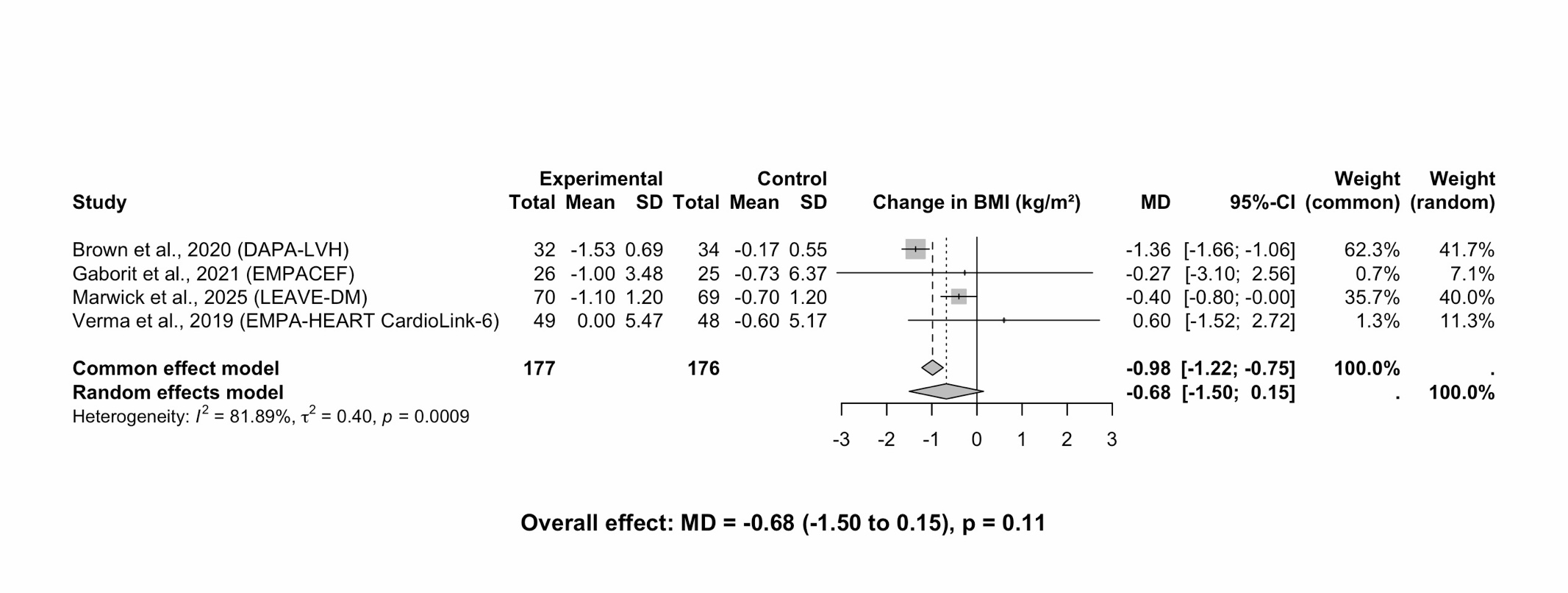


Supplementary Figure 11 : Sensitivity analysis for BMI excluding high-RoB study.

References

1. Brown AJM, Gandy S, McCrimmon R, Houston JG, Struthers AD, Lang CC. A randomized controlled trial of dapagliflozin on left ventricular hypertrophy in people with type two diabetes: the DAPA-LVH trial. European Heart Journal [Internet]. 2020 May 4;41(36):3421–32. Available from: https://doi.org/10.1093/eurheartj/ehaa419
2. Ersbøll M, Jürgens M, Hasbak P, Kjær A, Wolsk E, Zerahn B, et al. Effect of empagliflozin on myocardial structure and function in patients with type 2 diabetes at high cardiovascular risk: the SIMPLE randomized clinical trial. International Journal of Cardiac Imaging [Internet]. 2021 Oct 20;38(3):579–87. Available from: https://pubmed.ncbi.nlm.nih.gov/34669059/
3. Gaborit B, Ancel P, Abdullah AE, Maurice F, Abdesselam I, Calen A, et al. Effect of empagliflozin on ectopic fat stores and myocardial energetics in type 2 diabetes: the EMPACEF study. Cardiovascular Diabetology [Internet]. 2021 Mar 1;20(1):57. Available from: https://pmc.ncbi.nlm.nih.gov/articles/PMC7919089/#Sec15
4. Lim S, Bae JH, Oh H, Hwang IC, Yoon YE, Cho GY. Effect of ertugliflozin on left ventricular function in type 2 diabetes and pre-heart failure: the Ertu-GLS randomized clinical trial. Cardiovascular Diabetology [Internet]. 2024 Oct 22;23(1):373. Available from: https://doi.org/10.1186/s12933-024-02463-0
5. Lin J, Liu S, Liu T, Chuang S, Huang C, Chen Y, et al. ELUCIDATE Trial: a Single‐Center randomized controlled study. Journal of the American Heart Association [Internet]. 2024 Apr 19;13(9):e033832. Available from: https://doi.org/10.1161/jaha.123.033832
6. Marwick TH, Halabi A, Soh CH, Curtin A, Sherrif AG, Azad A, et al. Effects of dapagliflozin on the progression of left ventricular dysfunction in type 2 diabetes mellitus: a randomized controlled trial. Cardiovascular Diabetology [Internet]. 2025 May 30;24(1):232. Available from: https://doi.org/10.1186/s12933-025-02796-4
7. Rau M, Thiele K, Hartmann NUK, Schuh A, Altiok E, Möllmann J, et al. Empagliflozin does not change cardiac index nor systemic vascular resistance but rapidly improves left ventricular filling pressure in patients with type 2 diabetes: a randomized controlled study. Cardiovascular Diabetology [Internet]. 2021 Jan 7;20(1):6. Available from: https://doi.org/10.1186/s12933-020-01175-5
8. Shim CY, Seo J, Cho I, Lee CJ, Cho IJ, Lhagvasuren P, et al. Randomized, controlled trial to evaluate the effect of dapagliflozin on left ventricular diastolic function in patients with Type 2 diabetes mellitus. Circulation [Internet]. 2020 Nov 13;143(5):510–2. Available from: https://doi.org/10.1161/circulationaha.120.051992
9. Verma S, Mazer CD, Yan AT, Mason T, Garg V, Teoh H, et al. Effect of empagliflozin on left ventricular mass in patients with type 2 diabetes mellitus and coronary artery disease. Circulation [Internet]. 2019 Aug 22;140(21):1693–702. Available from: https://doi.org/10.1161/circulationaha.119.042375
10. Wang DD, Naumova AV, Isquith D, Sapp J, Huynh KA, Tucker I, et al. Dapagliflozin reduces systemic inflammation in patients with type 2 diabetes without known heart failure. Cardiovascular Diabetology [Internet]. 2024 Jun 7;23(1):197. Available from: https://doi.org/10.1186/s12933-024-02294-z
